# Pruning Brain Vasculature for Efficiency

**DOI:** 10.1371/journal.pbio.1001375

**Published:** 2012-08-14

**Authors:** Caitlin Sedwick

**Affiliations:** Freelance Science Writer, San Diego, California, United States of America

**Figure:**
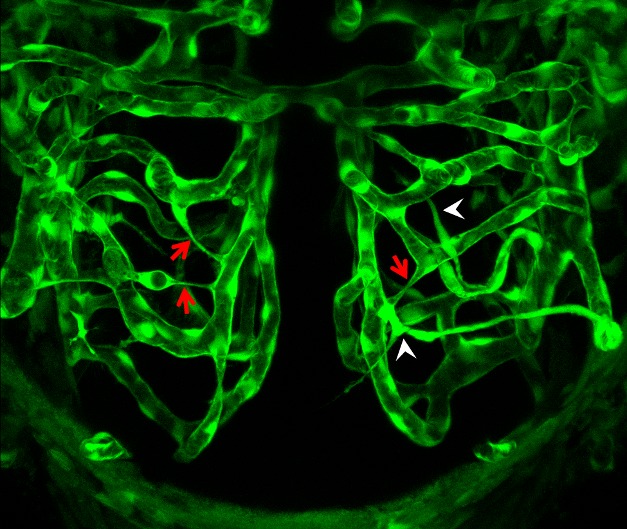
Developing brain blood vasculature undergoes not only angiogenic sprouting (arrowheads), but also extensive vessel pruning (arrows) driven by blood flow.

The brain is constantly integrating and analyzing information from myriad sources. All this activity requires a continuous flow of nutrients that, in higher vertebrates, is supplied to the brain through an elaborate network of blood vessels—the brain vasculature. But it's a fragile system; disruption or malformation of the brain vasculature can cause serious neurological problems. For that reason, it's important to understand how the brain vasculature is formed and maintained.

The earliest steps of brain vasculature formation involve new brain blood vessels sprouting off an existing vascular structure (the perineural vascular plexus) and beginning to invade the brain. However, little is known about how these early sprouts evolve into the elaborate loom of vessels and capillaries that supplies the energy needs of the fully developed brain. That's the problem Qi Chen, Luan Jiang, Jiu-lin Du and colleagues set out to address in their paper, published in this week's *PLoS Biology*.

For their studies, Qi and colleagues chose to work with zebrafish embryos, which are transparent and develop outside the mother's body, facilitating the observation of developmental processes. The authors selected a line of transgenic zebrafish in which neurons and vascular endothelial cells are each marked with different fluorescent labels. They monitored changes in brain vasculature by taking 3-dimensional snapshots of live animals' midbrain region throughout the first several days of embryonic development. Using this approach, the authors observed that the first new blood vessel sprouts appear in the midbrain by day one post-fertilization, and that by day two, an intricate network of vessels had formed within the midbrain. This network contained many looped structures: “O-shaped” structures where one blood vessel would branch apart and then rejoin with itself to form a structure resembling a traffic roundabout; and “H-shaped” arrangements, where a short blood vessel bridges two parallel ones. Over the next few days, the lengths of individual vessel segments within that network increased. Nonetheless, the degree of complexity and interconnectedness within the network significantly decreased at the same time.

This decrease in network complexity was achieved through conversion of O-shaped and H-shaped structures to simpler topologies. For example, the bridge might disappear from an H-shaped structure (leaving two parallel vessels), while O-shaped structures could change into a simple straight pipe. These alterations occurred over the course of 1–2 days, with certain vessel segments becoming thinner and then collapsing around their hollow lumens as they were pruned away.

Why and how does such pruning occur? The authors hypothesized that the affected blood vessels may be redundant and that pruning them increases the efficiency of blood flow within the brain. To investigate this possibility, Chen et al. employed a different line of transgenic zebrafish that expresses fluorescent labels on both vascular endothelial cells and on blood cells. Using these animals, the researchers were able to monitor the velocity and direction of blood flow throughout the midbrain.

As expected, pruning increased mean blood flow velocity and decreased the number of vessels exhibiting bidirectional blood flow. It also turned out that vessel segments that were pruned later generally had lower blood flow velocity than neighboring unpruned ones. These data suggest that low blood flow triggers vessel pruning—a conclusion that was also supported by experiments showing that pharmacologically increasing embryonic heart rate (which accelerates blood flow throughout the brain) reduces the level of pruning observed later on. Moreover, when blood flow through a particular vessel segment was blocked by injection of fluorescent micro-beads, pruning invariably eliminated that segment. Therefore, pruning likely serves to eliminate functionally redundant structures from the brain vasculature.

If this is the case, it raises the question of how pruning is brought about. One possible explanation is that the endothelial cells making up these segments die by apoptosis, as happens elsewhere during development. Alternatively, endothelial cells may simply withdraw from pruned segments and relocate. In fact, the authors did not find any evidence of apoptosis, but they observed that endothelial cells migrated away from pruned segments into the neighboring unpruned segments in most cases where vessels were “simplified”. Additionally, they observed that Rac1, a protein that is required for cell migration, is activated in the endothelial cells of the pruned segments. Finally, when the authors treated blood vessels with an inhibitor of Rac1, they observed a dramatic reduction in pruning.

Ultimately, these data point to a novel mechanism for vessel pruning—endothelial cell migration driven by Rac1—that is triggered by the regulation of blood flow within the brain. It will be interesting to see what other molecular mechanisms underlie this process, and whether it is employed in other developmental settings.

**Chen Q, Jiang L, Li C, Hu D, Bu J-w, et al. (2012) Haemodynamics-Driven Developmental Pruning of Brain Vasculature in Zebrafish. doi:10.1371/journal.pbio.1001374**

